# Supplementary figures and images for: PmbHLH58 from Pinus massoniana Improves Drought Tolerance by Reducing Stomatal Aperture and Inducing ABA Receptor Family Genes in Transgenic Poplar Plants
Source: Int J Mol Sci. 2024 Dec 31;26(1):277. doi: 10.3390/ijms26010277 (PMC11719964; doi:10.3390/ijms26010277)

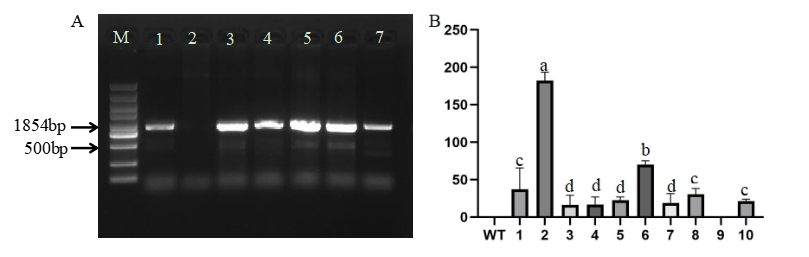

Supplement: Supplementary file 1 [file ijms-26-00277-s001.zip › Supplemental Figure 1.png]

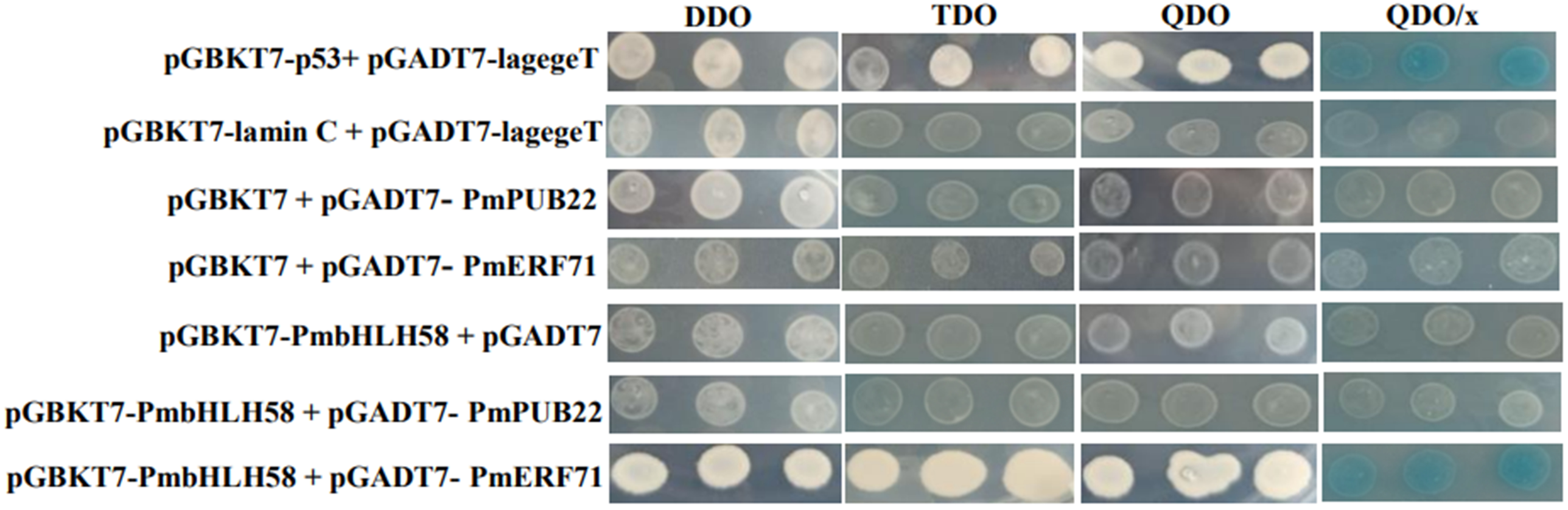

Supplement: Supplementary file 1 [file ijms-26-00277-s001.zip › Supplemental Figure 2.jpg]
